# Comparing the activity level of salivary matrix metalloproteinase‐8 in patients with diabetes and moderate to severe chronic generalized periodontitis

**DOI:** 10.1002/cre2.865

**Published:** 2024-03-03

**Authors:** Fatemeh Tavakoli, Masoumeh Faramarzi, Sepideh Salimnezhad, Bahare Jafari, Hosein Eslami, Bardia MohammadPourTabrizi

**Affiliations:** ^1^ Oral and Maxillofacial Medicine Department, School of Dentistry Shiraz University of Medical Sciences Shiraz Iran; ^2^ Department of Periodontology, Faculty of Dentistry Tabriz University of Medical Sciences Tabriz Iran; ^3^ School of Dentistry Tabriz University of Medical Sciences Tabriz Iran; ^4^ School of Dentistry Shiraz University of Medical Sciences Shiraz Iran; ^5^ Oral and Maxillofacial Medicine Department, School of Dentistry Tabriz University of Medical Sciences Tabriz Iran

**Keywords:** diabetes, generalized chronic generalized periodontitis, MMP‐8

## Abstract

**Objectives:**

The response of the host to plaque can be affected by systemic diseases like diabetes, hormonal changes, or immunological deficits, which can hasten the progression and severity of periodontitis. This study aimed to compare the activity of salivary matrix metalloproteinase‐8 (MMP‐8) in patients with moderate to severe generalized chronic generalized periodontitis between healthy individuals and those with type 2 diabetes who were referred to the Tabriz School of Dentistry.

**Materials and Methods:**

For this cross‐sectional study, 90 patients were randomly divided into three groups based on inclusion and exclusion criteria: patients with chronic generalized periodontitis with diabetes, patients with generalized chronic periodontal disease with normal blood glucose, and a control group of 30 healthy individuals. Participants were instructed not to brush their teeth for 12 h and not to eat or drink for 90 min before saliva sampling. Saliva samples were immediately stored at −80°C and analyzed using an ELISA test.

**Results:**

The results showed that there was a significant difference in salivary MMP‐8 levels among the three groups. Patients with periodontitis and diabetes had the highest levels of salivary MMP‐8, while the control group had the lowest levels. This indicates that chronic generalized periodontitis is strongly associated with the activity level of salivary MMP‐8, and elevated levels of MMP‐8 in diabetic patients demonstrate the impact of diabetes on periodontal disease.

**Conclusion:**

This study highlights the importance of monitoring salivary MMP‐8 levels in patients with periodontitis, especially those with diabetes. It also emphasizes the need for proper management of systemic diseases to prevent or slow down the progression of periodontal disease.

## 1 INTRODUCTION

Periodontitis is defined as a multifactorial chronic inflammatory disease characterized by the progressive destruction of the tooth‐supporting apparatus, including the gingiva, periodontal ligament, cementum, and alveolar bone, by the 2017 World Workshop on the Classification of Periodontal and Peri‐implant Diseases and Conditions (Caton et al., 2018). There are three prevalent kinds of periodontitis: chronic, invasive, and systemic. Chronic periodontitis, the most prevalent type, impacts over 30% of the population (Borgnakke et al., 2013). Untreated chronic periodontitis frequently leads to the formation of calculus, gingival abscesses, periodontal pockets, attachment loss, and bone resorption. The gingiva appears light to purple and exhibits mild to moderate swelling. The gingival borders may exhibit a rounded shape, whereas the papillae may appear flattened (Fiebig et al., 2008). Clinical periodontal disease severity has been developed by both the American Association of Periodontology and the European Federation of Periodontology. The methods used in the evaluation and analysis of periodontal disease in actual epidemiologic studies are varied, including probing pocket depth (PPD) only and combinations of PPD and bleeding on probing (BOP). Evaluation of the presence of buccal recessions with PPDs greater than 3 mm, if such recessions are present, the patient is a possible periodontitis case. If there is no buccal PPD greater than 3 mm, patients should be evaluated for full‐mouth BoP. If this is present in more than 10% of the sites, the patient is diagnosed with gingivitis and if present in less than 10% of sites, the patient is diagnosed with periodontal health (Eke et al., 2012; Tonetti et al., 2018). Tooth displacement occurs in cases of severe bone loss. Periodontal damage caused by chronic periodontitis typically intensifies with time. Age is associated with a progressive accumulation of deleterious effects that contribute to bone loss (Corrado et al., 2020). Systemic disorders such as diabetes impact the body's response to tooth plaque, accelerating periodontitis progression and increasing the severity and extent of periodontal tissue loss (Teeuw et al., 2010). Diabetes is a systemic disease with a significant impact on periodontitis, and hyperglycemia is a defining hallmark of diabetes. Reduced insulin production, altered insulin action, or both obstruct glucose access from the bloodstream into the tissues (Rahman et al., 2021). The HbA1c is now recommended as a standard of care (SOC) for testing and monitoring diabetes, specifically type 2 diabetes. HbA1c is the average blood glucose (sugar) levels for the last 2–3 months. An ideal HbA1c level is 48 mmol/mol (6.5%) or below (Prahalad et al., 2022). As a result, the amount of glucose in the blood and urine increases. Also, periodontal disease has been linked to the development of type 2 diabetes and even causes diabetes. Those with periodontitis and type 2 diabetes have more inflammatory mediators in their saliva and gingival crevicular fluid than nondiabetic periodontitis patients (Chapple & Genco, 2013). Studies show that treating periodontitis can improve control over blood sugar levels and reduce resistance to insulin in diabetic patients (Nana et al., 2021; Santonocito et al., 2022; Teeuw et al., 2010).

MMPs are among the most important inflammatory mediators, an essential group of 23 proteins that degrade the extracellular matrix (ECM) and are critical for tissue remodeling and degradation. MMP‐8 is one of the members of this family; it is also known as neutrophil collagenase and collagenase 2 (Nagase et al., 2006; Ortega et al., 2003; Xue et al., 2006). MMP‐8 breaks the triple helix structure of collagen during collagen decomposition (Murphy & Knäuper, 1997) and increases the expression of this factor in neutrophils during wound healing inflammation (Nwomeh et al., 1999). MMP‐8 expression can be increased to induce differentiation and osteoclast activity, causing tissue damage in the host.

Among all MMPs, MMP‐8 is known as a significant marker in chronic periodontitis, and it is possible to detect it in saliva and gingival crevicular fluid (Ramseier et al., 2009). Within gingival crevicular fluid, MMP‐8 was responsible for more than 90% of the collagenolytic activity. Hence, MMP‐8 is regarded as a highly prospective biomarker in oral fluids for the detection of periodontitis (Luchian et al., 2022). It has also been found that MMP‐8 levels drop during nonsurgical periodontal treatment. Additionally, MMP‐8 and its impact on the process of wound healing appear to be associated with hindered wound healing and other vascular issues in individuals with diabetes, which is a significant concern for public health (Hariono et al., 2018). Diabetes is a significant risk factor that has a detrimental impact on the tissues around the teeth, leading to an increased vulnerability to periodontitis. Moreover, the elevated concentrations of MMP‐8 in saliva unambiguously indicate the presence of active periodontitis in individuals with diabetes (Pussinen et al., 2007). In diabetic conditions, the failure of the healing process is exacerbated by the expression of these MMPs. In patients with chronic periodontitis, treatment strategies that target the inhibition of these MMPs may result in a quicker rate of recovery (Saremi et al., 2023; Yang et al., 2021). The purpose of this study was to compare the quantity of MMP‐8 in the saliva of patients with type 2 diabetes and periodontitis to the amount in the saliva of individuals with periodontitis but normal blood sugar levels. Diabetes and periodontitis are multifactorial diseases that may be affected by many factors, including the environment and race. In this study, we investigated the level of MMP‐8 in the saliva of people with periodontitis and diabetes in a population with different heredity, geographical factors, race, and so on.

## 2 METHODS

### 2.1 Study design

The target population is diabetic and nondiabetic patients with chronic generalized periodontitis who were diagnosed in the periodontics department. The current cross‐sectional study was conducted for 6 months in the Department of Oral Diseases and the Periodontology Department of Tabriz Dental School. To determine the sample size, the results of Gupta et al.'s study (2015) were used. The average level of MMP‐8 in the periodontitis group without diabetes was 380 (±80) and in the periodontitis group with diabetes was 440 (±80) and the control group was used. Considering the power of 80%, 30 samples were selected in each group, a total of 90 samples. After obtaining informed consent, they entered the study. Blood glucose is measured in mmol/L (millimoles per liter) or mg/dL (milligrams per deciliter). The normal range is between 4 and 6 mmol/L or 72 to 108 mg/dL (Mathew & Tadi, 2020). Criteria for the diagnosis of diabetes mellitus are symptoms of diabetes plus HbA1c ≥ 6.0% (Munjal et al., 2019).

### 2.2 Inclusion and exclusion criteria of the study group and control group

The inclusion criteria for the study group included:

– Having a history of type 2 diabetes.
– The average age of 40–60 years. It is necessary to explain that type 2 diabetes occurs in people over the age of 40.
– Suffering from chronic generalized periodontitis that more than 30% of the teeth have lost their adhesion.
– Willingness to participate in the study.

And for the control group was:

– Average age 40–60 years.
– Suffering from chronic generalized periodontitis that more than 30% of the teeth have lost their adhesion.
– Having normal blood sugar.
– Willingness to participate in the study.

The exclusion criteria for both groups included:

– Unwillingness to participate in the study.
– Having any systemic disease other than diabetes and debilitating diseases that can affect a person's oral health.
– Any nonsurgical periodontal treatments that have been performed during the last 9 months.
– Smokers or quit smoking for less than 5 years.
– During the 6‐month study period, there is a possibility of pregnancy or intention to become pregnant.

### 2.3 How to intervene

After selecting the patients according to the inclusion criteria and the standardization of diabetic and periodontal inflammation values had been ensured in the study groups, the samples were divided into three groups. The First group consisted of 30 patients with chronic generalized periodontitis with normal blood sugar, the second group consisted of 30 patients with chronic generalized periodontitis with diabetes, and the third group consisted of 30 healthy individuals as the control group. All three groups had saliva samples taken. The saliva sampling method was according to the Navazesh (1993) protocol. Participants were not allowed to brush their teeth for 12 h and eat or drink for 90 min before saliva sampling. Neither participant was taking any medication at the time of the study. The participants were given water to wash their mouths 15 min before sampling and then their oral cavity was checked to make sure that there was nothing in the oral cavity. Before sampling, the patients were asked to swallow their saliva. To collect saliva, participants were asked to spit 5 mL of their saliva in clean and dry polyethylene vials between 9 and 11 a.m. in a calm sitting position. All samples were immediately stored at −80°C and subjected to immunological analysis (ELISA) at the right time. A special ELISA kit was used to measure the amount of MMP‐8.

### 2.4 Statistical analysis method

SPSS 20 statistical software was used to analyze the data obtained from the study and data were expressed descriptively (prevalence, mean, median, and standard deviation). Kruskal–Wallis, Wilcoxon, and Mann–Whitney tests were used to compare the three groups in the study. In this study, a *p*‐value less than .05 was considered significant.

## 3 RESULTS

Ninety people were examined in this study. Thirty healthy people as control group, 30 patients with periodontitis without diabetes, and 30 patients with periodontitis and type 2 diabetes. Fifteen women and 15 men were examined in each group. Examining the age of patients in three groups showed that in the control group, the average age was 50.2 ± 7.08 years; in the group of patients with periodontitis and without diabetes, it was 50.7 ± 7.2 years; in patients with periodontitis and type 2 diabetes, it was equal to 48.46 ± 5.99 years. Analysis of variance (ANOVA) analysis of variance did not show a significant difference in the age of patients in the three studied groups.

The examination of blood sugar levels in patients of three groups showed that in the control group, the sugar level was 97.03 ± 5.55, in the periodontitis group without diabetes, it was 95.23 ± 5.84, and in the periodontitis group with diabetes, it was 183.4 ± 59.68. The Kruskal–Wallis test shows a significant difference in the amount of fasting blood sugar (FBS) of the three studied groups. The exact figures are shown in Table 1.

**Table 1:** Comparison of blood FBS of patients in three study groups.

	Mean	Standard deviation	Minimum	Maximum
Control	97.033	5.555	88.00	105.00
Periodontitis without diabetes	95.233	5.846	82.00	104.00
Periodontitis with diabetes	183.43	59.685	100.00	314.00
*p*‐Value	.000

*Note*: *p*‐Value: Kruskal—Wallis test.

Abbreviation: FBS, fasting blood sugar.

The average value of blood HbA1c (long‐term sugar) in periodontitis patients with diabetes was 9 ± 1.87% (Table 2). Examining the level of salivary MMP‐8 shows a significant difference between the three groups. The levels of salivary MMP‐8 in patients with periodontal disease and diabetes are significantly higher than those in the other two groups. In the periodontitis group without diabetes, the level of salivary MMP‐8 is significantly higher than the control group and lower than the group with periodontitis and diabetes. The lowest amount of salivary MMP‐8 was observed in the control group (Tables 3 and 4).

**Table 2:** Comparison of blood HbA1c (long‐term sugar) in periodontitis patients with diabetes.

	Amount	Mean	Standard deviation	Minimum	Maximum
HbA1c	30	9.00	1.875	6.00	12.00

**Table 3:** Comparison of salivary matrix metalloproteinase‐8 (MMP‐8) levels before and after periodontitis treatment in patients.

	Mean	Standard deviation	*p*‐Value
Control	205.00	14.431	
Periodontitis without diabetes	380.63	18.079	.000
Periodontitis with diabetes	450.60	69.272	.000
*p*‐Value	.000		

*Note*: *p*‐Value: Wilcoxon test and Kruskal–Wallis test.

**Table 4:** Two‐by‐two comparison of groups in terms of salivary matrix metalloproteinase‐8 (MMP‐8) levels.

I	J	Difference in means (I–J)	*p*‐Value	with a confidence level of 95%	
				Lower limit	Upper limit
Control	Periodontitis without diabetes	−175.63*	.000	−201.593	−149.673
Control	Periodontitis with diabetes	−245.60*	.000	−271.560	−219.639
Periodontitis without diabetes	Periodontitis with diabetes	−69.96*	.000	−95.926	−44.006

*Note*: *p*‐Value: Mann–Whitney test.

**Figure 1:**
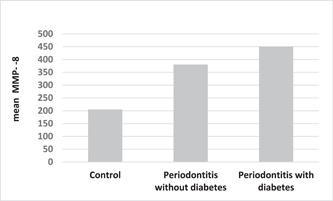
Comparison of salivary matrix metalloproteinase‐8 (MMP‐8) level in three groups.

Results show that:

– There is no significant relationship between the amount of FBS and salivary MMP‐8 levels in the control group.
– FBS and salivary MMP‐8 levels have a significant positive correlation among patients with periodontitis and without diabetes.
– FBS and salivary MMP‐8 levels have a significant positive correlation among patients with periodontitis and diabetes (Table 5) (Figure 1).

**Table 5:** Investigating the relationship between blood FBS and salivary matrix metalloproteinase‐8 (MMP‐8) levels in three groups.

			MMP‐8
FBS	Control	Correlation coefficient	.137
		*p*‐Value	.160
	Periodontitis without diabetes	Correlation coefficient	.395
		*p*‐Value	.031
	Periodontitis with diabetes	Correlation coefficient	.881
		*p*‐Value	.000

Abbreviation: FBS, fasting blood sugar.

## 4 DISCUSSION

Periodontal disease and diabetes are inflammatory diseases that cause changes in the level of various inflammatory factors. Studies in animals and humans agree that both forms of diabetes increase inflammatory events in periodontal tissue, and impair new bone formation; it also suggests that multiple cell types in periodontal tissues are affected by diabetes, including leukocytes, vascular cells, mesenchymal stem cells, periodontal ligament fibroblasts, osteoblasts, and osteocytes (Graves et al., 2020; Rad et al., 2006). Diabetes mellitus is a significant risk factor for developing periodontal diseases (Vernillo, 2001). At the same time, much evidence considers periodontitis as a risk factor for diabetes mellitus (Amiri et al., 2014). Periodontitis is defined as the sixth complication of diabetes, which means that diabetes can promote the progression of periodontitis. Conversely, periodontitis is now known as a risk factor for worsening glycemic control and may increase the risk for diabetic complications (Nguyen et al., 2020; Santonocito et al., 2022).

Diabetes is a metabolic condition that results in hyperglycemia because of flaws in insulin secretion, function, or both (Kashi et al., 2015). Diabetic patients suffer from periodontitis 2.8 times more often than healthy people and show 4.2 times more bone loss. Blood sugar levels have a direct correlation with the severity of periodontitis (Shiva et al., 2016). The gingivitis index and the level of attachment loss are higher in diabetic patients (Maboudi & Milani, 2016). Diabetes changes the response of periodontal tissue to local factors (Xiong et al., 2009).

Matrix metalloproteinases are proteolytic enzymes that break down the ECM and the basement membrane. In this sense, they are vital in physiological and pathological processes (Łukaszewicz‐Zając et al., 2013). The migration of lymphoid and myeloid cells, wound healing, and physiological tissue remodeling are greatly influenced by MMPs (Kessenbrock et al., 2010). Therefore, the measurement of this biomarker in saliva can be considered a noninvasive, easy, and inexpensive technique for diagnosis of the disease and a useful indicator for evaluating the disease's state (Baljani et al., 2023; Eslami et al., 2023; Srivastava et al., 2017). For this purpose, the present study was conducted with the aim of comparing salivary MMP‐8 levels in patients suffering from chronic generalized periodontitis with type 2 diabetes.

In this study, 90 patients were examined in three groups: Control group (30 people), periodontitis patients without diabetes (30 people), and periodontitis patients with diabetes (30 people). The abundance of women and men was the same in all three groups, and the average age was similar in the three groups. Nearly all of the participants had a similar sociodemographic status.

The levels of salivary MMP‐8 in the three groups were observed to be significantly different. For diabetic patients with periodontitis, the level of salivary MMP‐8 is considerably higher than for other groups. In the control group, it is considerably lower than in other groups.

In a study by Costa et al., it was demonstrated that MMP‐8 levels in saliva were remarkably higher in type 2 diabetic patients in comparison with those without type 2 diabetes Chronic generalized periodontitis was present for subjects of both groups (Costa et al., 2010). Chaparro et al. showed that during early pregnancy, women with severe periodontitis experience a rise in MMP‐8 and ‐9 concentrations, which is related to the onset of gestational diabetes mellitus (Chaparro et al., 2021). These researchers showed that periodontal indices (PI, GI, CAL, and PD) in patients with diabetes are at a higher level than in patients without diabetes. The results of the mentioned studies are consistent and confirm the results of the present study (Mirnic et al., 2022; Pirih et al., 2021).

Diabetes mellitus causes an increase in the severity of periodontal disease, which is associated with an increase in periodontal tissue's response to pathogenic microorganisms and alternate collagen metabolism. According to the studies and histological analysis of periodontal biopsies from patients with diabetes, the number of inflammatory cells is higher and the amount of collagen is lower compared to healthy people (Rad et al., 2006). Higher levels of MMP‐8 were found in the gingival tissue of diabetic patients with chronic generalized periodontitis, which indicates that in diabetic conditions, the healing process is not successful due to the expression of MMPs.

Therapeutic strategies to inhibit these MMPs can lead to the cure of patients with chronic generalized periodontitis (de de Morais et al., 2018). In a study, Rathnayake et al. measured salivary biomarkers for the diagnosis of systemic diseases. The level of salivary MMP‐8 was higher in diabetic patients after heart surgery and patients with diseases relating to muscles and joints. In epidemiological studies, saliva biomarkers can be used potentially for screening (Rathnayake et al., 2013). Mohamed et al. showed that the prevalence of periodontal pathogens and salivary MMP‐8 levels was not significantly affected by type 2 diabetes (Mohamed et al., 2016). Javed et al. stated that diabetes did not affect periodontal indices and salivary MMP‐8 (Javed et al., 2014).

The results of the studies of these researchers are contrary to the results of the present study. The cause of this problem can be related to the small size of the statistical sample, the severity of periodontitis, and the severity of diabetes in the patients. Also, the age and sex of the subjects are confounding factors in such studies. Al‐Majid et al. showed in a meta‐analysis review that salivary MMP‐8 has the potential to be a valuable diagnostic and preventive biotechnological tool in periodontal diseases (Al‐Majid et al., 2018). In a study, Pandruvada et al. showed that inflammatory cytokines of periodontitis can stimulate gingival fibroblasts to secrete MMP‐8, and that may stimulate the breakdown of the ECM and basement membrane. This can be one of the possible mechanisms of periodontitis (Pandruvada et al., 2016). Miller et al. showed that high levels of MMP‐8 (more than twice the control group) significantly increase the risk of periodontal disease (Miller et al., 2006). Zhang et al. conducted a meta‐analysis and found that patients with periodontitis had higher levels of salivary MMP‐8 compared to control group participants (Zhang et al., 2018). Noack et al. found that periodontally healthy individuals had lower salivary and serum MMP‐8 levels than those with periodontitis. Serum level was dose‐dependently correlated with salivary MMP‐8 and also significantly related to clinical parameters (Noack et al., 2017). Cumulative effects of changes in cellular response to local agents, disruption of tissue integrity, and changes in collagen metabolism undoubtedly play a significant role in the susceptibility of diabetics to infection and destructive periodontal disease (Bendinelli et al., 2013; Hadaegh et al., 2009; Janghorbani & Amini, 2012; Salminen et al., 2010).

Inflammatory mediators produced locally in periodontitis enter the systemic bloodstream and can cause insulin resistance. On the other hand, in diabetic people, the end products of glycosylation react with special receptors in the gingival cells and cause the production of the final inflammatory protein (Cecoro et al., 2020; Chopra et al., 2022; Wang et al., 2022). These markers are produced locally, then enter the person's circulatory system and induce or sustain a systemic inflammatory state that can lead to insulin resistance in cells and poor blood sugar control (Preshaw & Bissett, 2019). In the presence of gram‐negative bacteria, the destruction of periodontal tissues can occur due to the improper release of these biomarkers, both in terms of type and quantity (Aquino‐Martinez et al., 2020).

Various studies conducted in similar fields all emphasize the importance of the mutual influence of periodontitis and diabetes on each other. Since diabetes is considered a risk factor for periodontitis and worsens oral conditions in these patients, it is necessary to encourage diabetic patients to observe oral hygiene and refer them for examinations and appropriate treatment measures.

It is suggested that in future studies, the clinical effects of MMP‐8 reduction or increase on periodontitis can be examined by examining periodontal indices, and also the role of MMP‐8 specific drugs or antagonists on periodontal disease and blood sugar control be studied.

## 5 CONCLUSION

In this study, the level of salivary MMP‐8 was significantly higher among patients with periodontitis and diabetes than in patients with periodontitis who did not have diabetes and healthy individuals. Also in patients with periodontitis without diabetes, the level of salivary MMP‐8 is significantly higher than healthy people.

## AUTHOR CONTRIBUTIONS

**Fatemeh Tavakoli**: Data curation; investigation; validation. **Masoumeh Faramarzi**: Conceptualization; formal analysis; methodology; visualization. **Sepideh Salimnezhad**: Investigation; writing—original draft. **Bahare Jafari**: Investigation. **Hosein Eslami**: Conceptualization; methodology; project administration; supervision. **Bardia MohammadPourTabrizi**: Validation.

## CONFLICT OF INTEREST STATEMENT

The authors declare no conflict of interest.

## ETHICS STATEMENT

This study was approved by the ethics committee of Tabriz University of Medical Sciences (IR.TBZMED.REC.1397.517).

## Data Availability

All data generated or analyzed during this study are included in this published article.
